# Subclinical Detection of Diabetic Cardiomyopathy with MicroRNAs: Challenges and Perspectives

**DOI:** 10.1155/2016/6143129

**Published:** 2015-12-06

**Authors:** Luis E. León, Sweta Rani, Mauricio Fernandez, Martín Larico, Sebastián D. Calligaris

**Affiliations:** ^1^Centro de Genética y Genómica, Facultad de Medicina, Clínica Alemana Universidad del Desarrollo, 7710162 Santiago, Chile; ^2^Regenerative Medicine Institute (REMEDI), National University of Ireland, Galway, Ireland; ^3^Clínica Alemana, 7650568 Santiago, Chile; ^4^Centro de Medicina Regenerativa, Facultad de Medicina, Clínica Alemana-Universidad del Desarrollo, 7710162 Santiago, Chile

## Abstract

The prevalence of cardiac diabetic diseases has been increased around the world, being the most common cause of death and disability among diabetic patients. In particular, diabetic cardiomyopathy is characterized with a diastolic dysfunction and cardiac remodelling without signs of hypertension and coronary artery diseases. In an early stage, it is an asymptomatic disease; however, clinical studies demonstrate that diabetic myocardia are more vulnerable to injury derived by acute myocardial infarct and are the worst prognosis for rehabilitation. Currently, biochemical and imaging diagnostic methods are unable to detect subclinical manifestation of the disease (prior to diastolic dysfunction). In this review, we elaborately discuss the current scientific evidences to propose circulating microRNAs as promising biomarkers for early detection of diabetic cardiomyopathy and, then, to identify patients at high risk of diabetic cardiomyopathy development. Moreover, here we summarise the research strategies to identify miRNAs as potential biomarkers, present limitations, challenges, and future perspectives.

## 1. Introduction

The global prevalence of diabetes was estimated as 387 million people in 2014 and expected to increase to 592 million by 2035. Diabetes is a chronic disease that leads to multisystem complications such as nephropathy, retinopathy, neuropathy, and cardiovascular diseases. In 2014 alone about 4.9 million people died due to diabetes-related diseases, cardiovascular disease being the most common cause of death and disability among people with diabetes [1].

Diabetes slowly reduces heart function, (1) promoting the generation of atheroma in coronary arteries (atherosclerosis), reducing oxygen and nutrient supply to cardiac cells [2], (2) impairing autonomic nerve fibres that innervate the blood vessels and heart that produce abnormalities in control heart rate (arrhythmia) and vessels dynamics (cardiac autonomic neuropathy) [3], and (3) reducing contractile capacity of muscle cells and decreasing capillarity irrigation of myocardium (diabetic cardiomyopathy) [4]. While atherosclerosis is well known to increase the risk of heart failure through an episode of ischemia, the weakening of the cardiac fibres by the silent progression of diabetic cardiomyopathy is not accounted due to technical limitations in its subclinical detection. Population-based cohort studies reported by From et al. suggested that patients with heart failure and diabetes but no atherosclerosis had a higher risk of death [5]. In fact, Shah et al. reported that diabetes is related to a higher risk of heart failure hospitalization or death, independently of the left ventricular efficiency fraction levels in patients after a myocardial infarct event; that is, diabetic patients have less capacity to recuperate cardiac functions to normal levels with respect to nondiabetic patients [6].

Diabetic cardiomyopathy was defined first in 1972 as a heart failure without signs of coronary artery disease, hypertension, or valvular or congenital heart disease by Rubler et al. [7]. In spite of the differences in etiology and metabolic profile, many pathophysiologic features of this cardiomyopathy are shared by diabetes mellitus type 1 (DMT1) and diabetes mellitus type 2 (DMT2) [8].

Considering these common pathophysiological mechanisms, in a prediabetic and diabetic state, plasma level of free fatty acid (FFA) is increased, producing an augmented uptake, accumulation, and oxidation of FFA in the cardiomyocytes. The excess of FFA utilization generates a concomitant suppression of glucose oxidation by the indirect inhibition of the pyruvate dehydrogenase and a downregulation of glucose transporters 1 and 4 (*GLUT1* and* GLUT4*), establishing a metabolic derangement. The deficiency of glycolysis intermediates decreases the mitochondrial ATP synthesis by oxidative phosphorylation. To compensate this limitation and to attend the high ATP requirement of the heart, *β*-oxidation of FFA has a prominent role in increasing oxygen consumption and reactive oxygen species (ROS), which upregulates the uncoupling proteins expression to balance the proton transmembrane gradient needed for ATP synthesis [9]. The excessive ROS production stimulates apoptotic signals by ceramide generation and mitochondrial cytochrome c release [10]. Besides the deficiency of energy production, the excitation contraction coupling, essential for cardiac contraction, is altered by impaired intracellular Ca^+2^ handling [9].

The mitochondrial oxidative stress and “glycolated” tissue state produces an endothelial dysfunction and a proinflammatory microenvironment that stimulated the infiltration of macrophages and leukocytes that aggravates heart inflammation and tissue damage [9]. The remodelling of extracellular matrix is characterized by an interstitial and perivascular fibrosis and abnormalities in microvasculature [11]. In a latent subclinical period, metabolic disturbances and structural abnormalities lead to a diastolic dysfunction, which subsequently progresses to left ventricular hypertrophy, contractility reserve impairment, and, eventually, a systolic dysfunction [12].

Currently, therapy for diabetic cardiomyopathy is based on glycaemia control and hypoglycaemic drugs administration and changes in lifestyle (for DMT2), which delay the progression to heart failure but not revert it; however, therapy efficacy can be improved with earliest detection [13]. In this review we discuss the usefulness and limitations of the current methods used in the clinic to diagnose diabetic cardiomyopathy. We summarize the scientific evidences to propose miRNAs as new generation of biomarkers at subclinical stages of this disease by reflexing in biofluids the myocardial metabolic derangement before cardiac dysfunction.

## 2. Diagnosis of Diabetic Cardiomyopathy

Diabetic cardiomyopathy detection is a challenge in the clinical practice due to lack of any specific pathognomonic histologic changes or imaging characteristics. However, diastolic dysfunction and cardiac hypertrophy (measured by tissue Doppler echocardiography) in the absence of coronary artery disease and hypertension have been considered the two principal hallmarks to propose a diagnosis of diabetic cardiomyopathy in asymptomatic diabetic patients [14, 15].

Imaging diagnosis techniques were also used to detect myocardial metabolic changes in diabetic patients. McGavock et al. were able to detect the excessive storage of lipid in myocardium of patients with prediabetic stage using proton magnetic resonance spectroscopy, proposing this finding as an indicator of heart failure risk. However, they did not demonstrate a correlation between myocardial lipid content and cardiac function [16]. In addition, positron emission tomography was used to establish an association between myocardial metabolic derangement and early manifestation of diastolic function impairment with negative results [17].

Regarding serological biomarkers, natriuretic peptides and brain natriuretic peptide (BNP) in particular were proposed as suitable biomarkers for diastolic dysfunction in diabetic patients [18]. However, the utility of this molecule is controversial because plasma BNP rise is associated with excessive stretching of heart muscle cells, a condition associated with several cardiac diseases [19]. Troponins plasma concentration is associated with the magnitude of cardiomyocyte death, resulting in a biomarker of heart damage without any specificity of the cardiac disease etiology. In addition, troponins in plasma have a short half-life and are usually used to predict and establish heart failure [20].

At present, neither a laboratory test nor imaging techniques appear to be useful in diagnosing diabetic cardiomyopathy apart from diastolic dysfunction and to predict the risk of heart failure, excluding coronary artery disease, hypertension, or congenital heart failure [13]. Therefore, regarding the subclinical detection of diabetic cardiomyopathy, regulators of metabolic changes in the heart, as mentioned above, also present in biofluids, could be appropriate candidates as biomarkers. In the last 6 years, a large number of publications have been reported with promising results about the correlation of diseases manifestation and miRNAs (showing potential as a new class of biomarker) [21].

## 3. miRNAs Role in Diabetic Cardiomyopathy

As we previously described, changes in gene expression of key molecules involved in the pathogenesis of diabetic cardiomyopathy can be influenced by environmental factors, for instance, high fat diets, tobacco smoke, or epigenetic factors [22]. At present, epigenetic studies describe three mechanisms to link the type of exposure with cellular gene expression response: DNA methylation, histone modification, and miRNA expression [23].

MicroRNAs or miRNAs are small noncoding RNA molecules (≈22 nucleotides) which downregulate gene expression by a posttranscriptional mechanism controlling approximately 30% of all protein-coding genes of mammalian genome [24, 25]. During the past 7 years, researchers have identified several miRNAs and their specific mRNA targets altered in diabetic cardiomyopathy using experimental models at preclinic level, demonstrating the significant role of miRNAs in the progression of diabetic heart complication (Table 1). Human biopsies of diabetic heart showed an upregulation of miR-223 with an inhibition of* GLUT4* gene expression, reducing glucose uptake. This miRNA-mRNA interaction was confirmed using neonatal rat cardiomyocyte [26]. El Azzouzi et al. reported that miR-199a/miR-214 cluster downregulated the peroxisome proliferator-activated receptor *δ* gene expression, which is a critical regulator of energy metabolism switch between fatty acid oxidation and glycolysis, impairing mitochondrial fatty acid oxidation [27]. Cardiomyocyte hypertrophy induced by exposing neonatal rat cardiomyocytes to high levels of glucose identified significantly reduced expression of three miRNAs (miR-150, miR-133a, and miR-373) involved in cardiac hypertrophy process [28–30]. Diao et al. identified sixteen microRNAs differentially expressed in hearts of DMT1 animal model induced by streptozotocin and proposed 4 gene targets (*Rasa1*,* Rac1*,* Tgfb3*, and* Col1A1*) associated with cardiac hypertrophy and myocardial fibrosis [31]. Upregulation of miR-34a and miR-1, induced by high glucose exposure, decreased* Bcl-2* and* Igf-1* gene expression, respectively, promoting apoptosis in H9c2 cells [32, 33]. Regarding cardiomyocyte Ca^+2^ handling during contractility-relaxation cycle, Yildirim et al. demonstrated that myocardial miR-1 downregulation produces an increase of junctin levels in streptozotocin-induced diabetic mice. Junctin is a component of the ryanodine receptor Ca^+2^ release channel complex in sarcoplasmic reticulum [34]. These data suggest that miR-1 has many target genes (e.g.,* Igf-1* and* Junctin*) and its regulation could depend on the experimental model, indicating the nonspecificity of this miRNA in diabetic cardiomyopathy. Upregulation of miR-30d promoted cardiomyocyte pyroptosis (the proinflammatory programmed cell death) in a DMT1 animal model, via the repression of foxo3a and apoptosis repressor with caspase recruitment domain expression and, consequently, the activation of caspase-1 and secretion of proinflammatory cytokines (IL-1*β* and IL-18) [35]. Using myocardial microvascular endothelial cells from a nonobese DMT2 animal model (Goto-Kakizaki rat), upregulation of miR-320 was reported that reduced* Igf-1* gene expression decreasing angiogenic response to diabetes-derived microvascular injury [36]. miR-301 upregulation alters the voltage-gated potassium channel in diabetic heart of* db/db* mice (animal model for DMT2), generating an electrical remodelling [37]. miR-141 upregulation in heart of diabetic mice (induced with streptozotocin) reduced the gene expression of* Slc25a3* (inner mitochondrial phosphate transporter), resulting in a decreased ATP production [38]. Recently, Kuwabara et al. showed that miR-451 exacerbates lipotoxicity in cardiac myocytes and cardiac hypertrophy in obese mice fed with high fat diet for 20 weeks (a physiological obese animal model for a prediabetic state of DMT2) through the direct interaction with Cab39, scaffold protein of liver kinase B1 (LKB1), which suppress the LKB1/AMPK pathway. As AMP-activated protein kinase (AMPK) is a major cellular response of energy availability, its suppression reduced the cardiac functional reserve [39]. We summarised the miRNAs in pathological mechanism of diabetes cardiomyopathy in Table 1.

## 4. miRNAs as Biomarkers

Besides the intracellular functions of miRNAs, recent studies demonstrated that miRNAs are also paracrine mediators of cell-to-cell communication transported via microvesicles called exosomes. Cardiac cell communication via exosomes in healthy and pathological conditions is an emerging research field for understanding the development of cardiac diseases [40]. Malik et al. published that oxidative stress or hypoxia/reoxygenation transition stimulated cardiomyocyte to secrete exosomes containing mRNAs and miRNAs [41]. In addition, external cell signalling, as growth factor stimulation, can regulate the transcriptional contents of secreted exosomes in cardiomyocytes [42]. Waldenström et al. reported that exosomes released from cardiomyocytes affect gene expression in fibroblast [43]. Reciprocally, cardiac fibroblast was found to release miR-21 via exosomes and was associated with cardiomyocyte hypertrophy [44]. According to the current scientific evidences, both miRNA composition and quantity could be considered a reflex of metabolic or differentiated state of exosome-producing cells. Circulating exosomes can be identified in all biofluids, particularly in plasma or serum [45], and, therefore, becoming an attractive tool for analytical studies and subsequent diagnosis of the diseases.

Moreover, circulating miRNAs in plasma are also transported and delivered to recipient cells on circulating high-density lipoprotein (HDL) [46]. HDL has a crucial role in the progression of cardiolipid-metabolism modifications occurring in diabetic cardiomyopathy development; for instance, in cardiomyocyte FFA oxidation via AMP-kinase activation and its accumulation prevents lipotoxicity in diabetic heart [47]. The discovery of miRNA participation in the regulation of lipoprotein synthesis, composition, transport, and degradation has provided new targets for therapy to improve the cardioprotective properties of HDL, particularly in coronary artery disease due to HDL regulation of cholesterol homeostasis [48]. Taking that into account, circulating miRNA in HDL complex could also be an indicator of metabolic changes in lipid-tissue accumulation diseases such as diabetic cardiomyopathy.

A third class of circulating miRNAs bind to soluble proteins called Argonautes, which are key players in all small-RNA-guided gene silencing processes [49]. Biophysics studies demonstrated that extracellular miRNA circulating in the bloodstream is remarkably stable, in spite of being presented in an RNAse-rich environment, due to its encapsulation in microvesicles/exosomes or its binding to the proteins [50]. This known stability of miRNA-exosomes and miRNA-protein complex is another relevant characteristic suitable for biomarkers.

## 5. Evidences of miRNA Profiling for Diabetic Cardiomyopathy

Identification of miRNA associated with diabetes and its complications in humans escalated with the possibility of screening multiple miRNAs simultaneously using profiling techniques including microarray and Next Generation Sequencing (NGS), in damaged or injury tissues of diabetes animal models due to miRNA-mRNA interaction found to be conserved between most mammalians [51]. The gene target identification of the selected miRNAs has been studied* in vitro* using neonatal rat cardiomyocytes where the miRNA-mRNA interaction is correlated with changes in cell phenotype. The process of gene targets identification is accelerated by bioinformatics techniques such as TargetScan, DIANA-mirExTra, PITA, miRNADA, miRDB, and PICTAR that reduce the number of possibilities to test experimentally [52–54]. In this context a good resource that integrates all these tools is miRWalk database that allows choosing between predicted and validated experimental miRNA-mRNA interactions [55].

The discovery of placental miRNA in maternal plasma in 2008 [56], along with the nascent hypothesis of its role in the cell-to-cell communication, has promoted the study of miRNAs as biomarkers in cancer through the specific secretion via exosomes by tumour cells [21]. In the same year, Lawrie et al. reported elevated levels of tumour-associated miRNAs in serum of patients suffering from diffuse large B-cell lymphoma [57]. Several clinical studies are also performed proposing use of miRNA in early diagnosis of chronic illness including cardiovascular and neurodegenerative diseases [58–60]. Therefore, pharmaceutical companies are very interested in developing a diagnostic kit for several types of cancer and chronic diseases [61].

According to the last version (21st) of miRNA database (miRBase) [62], there are 1881 human sequences identified and the list is still growing; Friedländer et al., employing an innovative computational method, reported 2469 novel human miRNA candidates [63]. Therefore, researchers have used two experimental strategies to find the** “**needle in a haystack”: using omics approaches (microarray or NGS) and/or preselecting miRNAs based on previous finding reported in animal models (Table 2). In this context, NGS offers several advantages; for instance, it does not require the knowledge of either miRNA target or specific probes or primers to discover new miRNAs [64]. Regarding diabetes, since 2010 several differentially regulated miRNAs have been identified in human biofluids from patients of impaired glucose tolerance (IGT)/impaired fasting glucose (IFG), DMT2 in comparison to healthy controls (Table 2).

Reviewing the articles published in the last five years, we have concluded that there is not an extensive overlap in the results of miRNAs identification associated with different conditions of diabetes patients. Only miR-126 and miR-144 have been proposed as biomarkers for diagnosing diabetes in more than one study [65–68]. Although these clinical studies used the same range of values for glucose tolerance test (the most important parameter for patients classification), there are other factors that could explain the differences: (1) clinical characteristics including obesity, age, year after diagnosis, and lipid profile [69]; (2) treatment with hypoglycaemic drugs such as metformin [66]; (3) ethnical origin such as Iraqis versus Swedes [70]; (4) technical aspects: types of biofluids, differences in miRNA microarrays companies (quantity and type of miRNA tested), and miRNA normalization methods.

Regarding diabetic cardiomyopathy, diagnosed patients with diastolic dysfunction share many clinical characteristics with diabetic patients reported in the clinical study for the identification of miRNAs related to diabetes (Table 2). However, at present there are no clinical trials reporting circulating miRNAs as a candidate for diabetic cardiomyopathy diagnosis. On the other hand, there is no concordance between the miRNA identified with diabetic cardiomyopathy in animal models and those identified in human biofluids, except miR-34a and miR-30d, which were identified first in human plasma and then their mechanism of action was studied at a preclinical level [32, 35]. The diabetic complications have a slow but progressive negative manifestation in the target organs (kidney, liver, heart, and retina) with respect to the apparent stability of metabolic parameters (fasting glucose and glucose tolerance test). As diabetes is a multifactorial disease, care should be taken when enrolling the patients, by following strict definitions of the clinical characteristic of the diabetic complications. In a cross-sectional study where DMT1 patients were classified in three groups according to their level of renal dysfunction by eGFR, good renal function ≥ 30 mL/min of creatinine clearance, renal failure < 30 mL/min of creatinine clearance, and healthy control, four miRNAs (miR-181, miR-326, miR-126, and miR-573-3p) were identified in plasma that could be useful to predict the development of diabetic nephropathy [71].

The use of disease animal models is a powerful tool to select circulating miRNAs candidate for biomarker as it allows establishing a correlation between miRNAs associated with specific injured organ and the biofluid from early to advanced stages of disease development. For instance, Bellinger et al. found a concordance between the expression of miR-714, miR-1188, miR-1897-3p, miR-877, and miR-1224 and progression of acute kidney injury reflected in the plasma of a mouse model, proposing them as a promising predictor of kidney injury [72]. Acharya et al. reported serum miRNA signatures that predict the impact of radiation in animals that were exposed to sublethal and lethal doses of radiation, 24 hours after exposure [73], and Rotkrua et al. selected circulating miRNAs (miR-103, miR-107, miR-194, and miR-210) as biomarkers for early detection of diffuse-type gastric cancer using a mouse model and compared expression of these miRNAs in tumour tissue and serum samples [74]. The strategy of using diabetic cardiomyopathy animal models to find a corelation of miRNAs expression between myocardium and biofluids would not only provide relevant information but also accelerate miRNA identification [8, 75].

Regarding methodological aspects, it has been established that using different evaluation techniques may yield variations in the end results. For this reason, in the last years many methodological studies have been published comparing different laboratory procedures: (1) sample collection, (2) total miRNA isolation, (3) miRNA profiling methods, including qRT-PCR, miRNA microarrays (GeneChip and miRCURY LNA), and NGS, and (4) criteria of data analysis including miRNA normalization method (spike-in or internal miRNA). Further, alternatives in miRNA procedures were elaborately discussed in an excellent review of Moldovan et al. [76].

**Figure 1:**
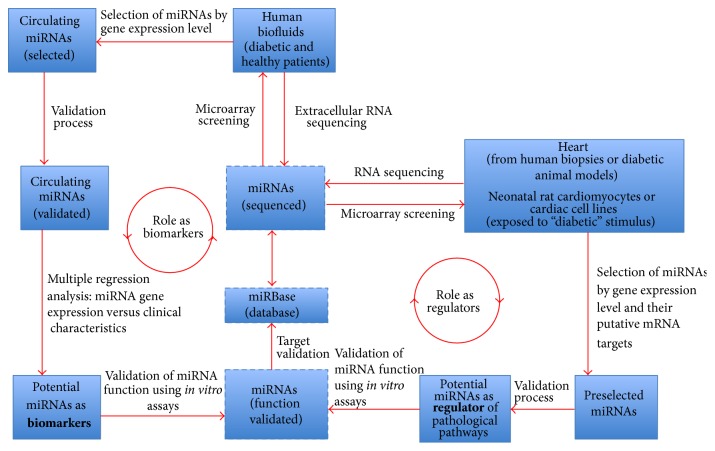
Experimental strategies for circulating microRNA identification as potential biomarkers.

In a future perspective, a consistent miRNA profiling is not enough to diagnose a disease; informatics algorithms such as naïve Bayes classifier, J48 Decision Trees, and support vector machines are also necessary to identify the best miRNA profiling (considering all clinical characteristics) to discriminate between diabetic patients, which of them have a high risk of developing diabetic cardiomyopathy. A summary of the experimental strategies for the identification of circulating miRNAs as biomarkers is described in Figure 1.

## 6. Conclusions

Diabetic cardiomyopathy progresses slowly and silently and is diagnosed (with the current detection procedures) only when the heart manifests a certain grade of dysfunction. This subclinical state of the illness becomes even more critical after an ischemic episode as it reduces the possibility to rescue the heart function to normal levels. At present, scientific evidence of the potential use of circulating miRNAs as biomarkers for cardiovascular diseases is increasing every day and is extensively backed up by the studies carried out by research groups of public and private institutions. The identification of miRNAs in diabetic patients “without” any secondary complications is relevant as it would allow narrowing down the miRNAs associated with specific diabetic complication. Regarding diabetic cardiomyopathy, the early detection using miRNAs biomarker could serve to intensify the antidiabetes treatment and cardioprophylactic therapies in patients with high risk of diabetes-derived diastolic dysfunction before it appears.

## Figures and Tables

**Table 1:** Identification of miRNA involved in diabetic cardiomyopathy pathogenesis.

miRNA	Gene expression	Preselected miRNAs/screening method	Tissue source/experimental model	Target genes and/or pathophysiological effect	References
miR-1	↑	miRNA selected from [77, 78]	H9c2 cells exposure to high glucose levels	Block IGF-1 signal pathway inducing apoptosis	[33]

miR-1	↓	miR-1, miR-21, miR-133a, miR-499, miR-133b/1900 microRNAs approx. GeneChip miRNA arrays based on miRBase 17, Affymetrix	Mice heart/STZ-induced diabetic rat for 5 weeks (1 dose of 50 mg/Kg)	Junctin, which is involved in cardiomyocyte calcium handling	[34]

miR-320	↑	let-7e, miR-129, miR-291-5p, miR-320, miR-327, miR-333, miR-363-5p, miR-370, miR-494, miR-503, miR-664/274 miRNAs, microarray for miRNA based on miRBase 8, Exiqon	Myocardial microvascular endothelial cells/nonobese DMT2 animal model (Goto-Kakizaki rat)	IGF-1; angiogenic factor	[36]

miR-133a	↓	miR-1, miR-9, miR-16, miR-20, miR-23b, miR-24, miR-26a, miR-30a-5p, miR-30d, miR-93, miR-122a, miR-133a/b, miR-146a/b, miR-187, miR-197, miR-203, miR-207, miR-297, miR-299-5p, miR-320, miR-324-3p, miR-326, miR-335, miR-341, miR-345, miR-346, miR-62, miR-369-5p, miR-370, miR-371–miR-374, miR-422b, miR-431, miR-432, miR-467m, miR-483, miR-487a, miR-497, miR-500, and miR-518d/miRVana microarray for 486 miRNAs, Ambion microarray	Mice heart/STZ-induced diabetic mice for 2 months (1 dose of 150 mg/Kg)	Cardiac hypertrophy	[29]

miR-223	↓	TaqMan MicroRNA Assays Human Panel Early Access, for 155 different miRNAs, Applied Biosystems	Human heart/biopsies of NGT and DMT2 patients	GLUT4; glucose uptake	[26]

miR-373	↓	miR-1, miR-20a, miR-21, miR-24, miR-29, miR-142-3p, miR-143, miR-195, miR-199a-3p, miR-220b, miR-208a, miR-221, miR-373, miR-499-3p, miR-700, miR-705/CapitalBio Mammalian miRNA Array V4.0, based on miRBase 12, CapitalBio Corp.	Mice heart/STZ-induced diabetic mice for 2 months (1 dose of 150 mg/Kg)	Cardiac hypertrophy and myocardial fibrosis via mitogen-activated-protein kinase cascades pathway activation and RASA1, RAC1, TGFB3, and COL1A1 expression	[30, 31]

miR-141	↑	miR-141, miR-200c, miR-208b, miR-295/RT-PCR Array system for 376 miRNAs, SABiosciences	Mice heart/STZ-induced diabetic mice for 5 weeks (5 doses daily of 50 mg/Kg)	Slc25a3: regulator of the mitochondrial phosphate carrier expression, which is involved in ATP mitochondrial production	[38]

miR-30d	↑	miRNA selected for diverse papers reviewed in [35]	Rat heart/STZ-induced diabetic rat for 3 days (3 doses of 35 mg/Kg/day); neonatal rat cardiomyocyte exposure to high glucose levels	FOXO3a; induction of cardiomyocyte pyroptosis and cardiac inflammation	[35]

miR-34a	↑	miRNA selected from [79]	H9c2 cells exposure to high glucose levels	BCL-2; induction of apoptosis	[32]

miR-150	↓	miRNA selected from [80]	Neonatal rat cardiomyocyte exposure to high glucose levels	P300, which plays a role in cardiomyocyte hypertrophy	[28]

miR-301a	↑	miRNA selected from [81, 82]	Mice heart/db/db mice of 13-14 weeks of age	Kv4.2 channel; electrical remodelling	[37]

miR-451	↑	1300 miRNAs approx. Based on miRBase 19, miRNA microarray system 3D-Gene	Mice heart/obese mice fed with high fat diet for 20 weeks; neonatal rat ventricular cardiomyocytes exposure to palmitic acid	Cardiac hypertrophy through suppression of the LKB1/AMPK pathway	[39]

H9c2 cells: cardiac cell line derived from rat myocardium, STZ: streptozotocin, db/db mice: homozygous for diabetes spontaneous mutation in leptin receptor, NGT: normal glucose tolerance, and DMT2: diabetes mellitus type 2.

**Table 2:** Identification of miRNAs identified in human biofluids as potential biomarkers for diabetes mellitus type 2.

Proposed miRNAs as biomarkers	Gene expression (diabetic versus healthy patients)	Type of samples/normalized method	Preselected miRNAs from screening method or from preclinical studies	Type of clinical study/experimental groups (number)	Geographic location	References
miR-126	↓	Plasma/RT-qPCR, miR-454	miR-15a, miR-20b, miR-21, miR-24, miR-29b, miR-126, miR-150, miR-191, miR-197, miR-223, miR-320, miR-486/Human TaqMan MicroRNA Arrays for 377 miRNAs, Applied Biosystems	Prospective population-based study/NGT (580) IFG-IGT (162) DMT2 (80)	Bruneck, Italy	[65]

miR-144	↑	Blood/RT-qPCR	miR-144, miR-146a, miR-150, and miR-182 selected from blood liver, pancreas, skeletal muscle, and adipose tissue of rat fed with high fat diet (2 weeks) and STZ administration (40 mg/Kg, ip)/microarray for miRNA based on miRBase 11, Exiqon	Cross-sectional study/NGT (15) IGT-IFG (14) DMT2 (21)	Singapore, Singapore	[68]

miR-9 miR-29a miR-30d miR-34a miR-124a miR-146a miR-375	↑	Serum/RT-qPCR RNU6B	miRNAs selected from [83]	Cross-sectional study/NGT (19) IGT-IFG (19) DMT2 (18)	Jinan, China	[79]

miR-126 miR-140-5p miR-142-3p miR-195 miR-423-5p	↓	Plasma/RT-qPCR miR-106a miR-146a miR-19b miR-223	miR-125b, miR-126, miR-130b, miR-140-5p, miR-142-3p, miR-192, miR-195, miR-222, miR-423-5p, and miR-532-5p/TaqMan Array Human MicroRNAs v2.0, for 377 miRNAs, Life Technologies	Pilot study/NGT (6) DMT2 (6) Cross-sectional study/NGT (45) DMT2 (48)	Girona, Spain	[66]

miR-138, miR-376a miR-503	↓	Serum/RT-qPCR miR-191 miR-423-3p	miR-15b, miR-25, miR-27b, miR-101, miR-138, miR-150, miR-205, miR-376a, miR-432-5p, miR-500a, miR-503, and miR-942/Human Panels I and II containing 742 miRNAs, Exiqon	Cross-sectional study/NGT (20) Obese (20) DMT2 (13) Obese + DMT2 (16)	Madrid, Spain	[69]

miR-21 miR-210 (plasma) miR-126 (urine)	↑ ↓	Plasma, urine/RT-qPCR	miRNA-21, miR-126, and miR-210/miRNAs selected from [65, 84, 85]	Cross-sectional cohort study with paediatric patients NGT (79) DMT1 (68)	London, United Kingdom	[86]

miR-126	↓	Serum/RT-qPCR Cel-miR-39	miRNA selected from [65]	Cross-sectional cohort study/NGT (138), IGT/IFG (157), DMT2 (160)	Harbin, China	[67]

miR-24 miR-29b miR-144 (for Swedes)	↑	Plasma/RT-qPCR miR-425	miR-15b, miR-20, miR-21, miR-24, miR-28-3p, miR-29b, miR-126, miR-144, miR-150, miR-191, miR-197, miR-223, and miR-302a, miR-486-5p selected according to miRNA described in [65, 68]	Cross-sectional study/NGT-Iraq (65) NGT-Sweden (54) DMT2-Iraq (19) DMT2-Sweden (14)	(Sweden and Iraqi population) Malmö, Sweden	[70]

miR-101 miR-375 miR-802	↑	Serum/RT-qPCR Cel-miR-39	miR-101, miR-335, miR-375, and miR-802 selected by preselected miRNAs from heart, pancreas, white adipose tissue, and other tissues of obese mice fed with high fat diet of 20 weeks of age were obtained by sequence analysis Illumina	NGT (49) DMT2 (155)	Okayama, Japan	[87]

NGT: normal glucose tolerance, IGT-IFG: impaired glucose tolerance-impaired fasting glucose, DMT2: diabetes mellitus type 2, STZ: streptozotocin, and ip: intraperitoneal.
